# A Comparative Study of the Effects of Valproate and Oxcarbazepine on Sexual Function, Sperm Quality, and Sex Hormones in Males with Epilepsy

**DOI:** 10.1155/2021/6624101

**Published:** 2021-07-03

**Authors:** Yi Guo, Lang Chen, Dongmei Wu, Liang Yu, Hongbin Sun, Qiong Zhu

**Affiliations:** ^1^Department of Neurology, Sichuan Provincial People's Hospital, University of Electronic Science and Technology of China, Chengdu, China; ^2^Chinese Academy of Sciences Sichuan Translational Medicine Research Hospital, Chengdu, China; ^3^Southwest Medical University, Luzhou, China; ^4^Dazhou Central Hospital, Dazhou, China

## Abstract

**Aims:**

Although several studies have indicated that valproate (VPA) and oxcarbazepine (OXC) cause reproductive endocrine disorders and sexual dysfunction, there remains some controversy regarding these issues in males with epilepsy. This study is aimed at evaluating the effects of VPA and OXC on sexual function, sperm quality, and sex hormones in young males with epilepsy.

**Methods:**

Males with newly diagnosed epilepsy treated with VPA and OXC were recruited, and sexual function questionnaires (International Index of Erectile Function-5 (IIEF-5)), sperm quality, and sex hormone levels were assessed before treatment and at 6 months after treatment with VPA or OXC monotherapy.

**Results:**

Forty-four young males with epilepsy (23 treated with VPA, 21 treated with OXC) and 30 age-matched healthy individuals were recruited for our study. The sexual function, sperm quality, marriage rate, and fertility rate of these young males with epilepsy were lower than those of healthy controls. Sperm quality were significantly reduced in young male patients after 6 months of VPA administration. The level of follicle stimulating hormone (FSH) was increased in patients after OXC treatment. Meanwhile, sexual function and sperm quality were not affected.

**Conclusion:**

Sexual function and sperm quality were reduced in young males with epilepsy. VPA may exert a negative effect on sperm quality, whereas OXC has no harmful effect on sexual function and sperm quality in young males with epilepsy.

## 1. Introduction

Epilepsy is a common chronic neurological disorder that affects nearly 50 million people worldwide [1]. Approximately 60% of people with epilepsy can achieve seizure control with antiseizure medication (ASM) monotherapy [2]. An increasing number of studies have demonstrated that valproate (VPA) and oxcarbazepine (OXC) continue to be commonly used ASMs for patients with epilepsy [3, 4]. Meanwhile, concerns have been raised by patients and clinicians regarding the risk of sexual dysfunction, reduction of sperm quality, and sex hormone disorders in males with epilepsy [5, 6].

As a broad-spectrum ASM, an increasing number of studies have demonstrated that VPA has adverse effects on the reproductive system in females [7]. Limited evidence has also shown that VPA can lead to a significant reduction in sexual function and sperm quality and sex hormone disorders in males with epilepsy [8]. However, controversy remains regarding these effects of VPA [9–11]. To date, limited evidence showed that switching from other ASMs to OXC may improve sexual function in male patients [12]. However, little is known about the effect of OXC monotherapy on sexual function, sperm quality, and sex hormones in males with epilepsy. We performed a perspective study to evaluate the effects of VPA and OXC monotherapy on sexual function, sperm quality, and sex hormones in males with newly diagnosed epilepsy. In our study, we found that the sexual function, sperm quality, marriage rate, and fertility rate of male epilepsy patients were lower than those of controls. Sperm quality were significantly reduced in young male patients after 6 months of VPA monotherapy. However, sexual function and semen quality were unchanged in young male patients after 6 months of OXC monotherapy.

## 2. Materials and Methods

### 2.1. Study Population

The subjects in our study were newly diagnosed male epilepsy patients who were treated at the Epilepsy Center of Sichuan Provincial People's Hospital, China, from April 2015 to November 2016. Epilepsy was defined according to the diagnostic criteria formulated by the International League Against Epilepsy (ILAE) in 2014 [13]. Healthy male volunteers were recruited during the same period to comprise the control group. The exclusion criteria were as follows: (1) use of hormones, antidepressants, or drugs to improve sexual function; (2) patients with liver or kidney dysfunction, thyroid disease, diabetes, infectious diseases, varicocele, cryptorchidism, a history of testicular surgery, Klinefelter syndrome, or urinary system diseases; (3) patients who had a history of long-term alcoholism, smoking, or exposure to toxic substances; and (4) patients with mental illness, intracranial occupying lesions, brain injury, or progressive degeneration of the nervous system. Individual participation in the study was terminated if any of the following conditions were met: (1) Patients experienced adverse drug reactions after taking the medications and needed to discontinue the drug. (2) Patients failed to achieve an ideal treatment effect, and other antiepileptic drugs (AEDs) were substituted or added. (3) Patients or volunteers took medications or suffered from diseases that might affect the results during the study. (4) Participants withdrew from the study for personal reasons. The institutional review boards of Sichuan Academy of Medical Sciences and Sichuan Provincial People's Hospital provided ethical approval for this study, and written informed consent forms were signed by all participants.

### 2.2. Epilepsy Treatment and Data Collection

Patients with epilepsy were randomly treated with VPA (trade name: Depakote; packing specification: 500 mg × 30 tablets; manufacturer: Sailof (Hangzhou) Pharmaceutical Co., Ltd.; batch number: H2O010595) or OXC (trade name: Trileptal; packing specification: 0.3 g × 50 tablets; manufacturer: Novartis Farma S.P.A. (Italy); batch number: H2O130016). The dosage of VPA was initially 250 mg twice daily and then gradually increased to an effective dose; the maximum daily dose did not exceed 30 mg/kg. The dosage of OXC was initially 300 mg twice daily and then gradually increased to an effective dose; the maximum daily dose did not exceed 2400 mg/day. All patients were followed up once a month by a physician.

A sexual function questionnaire survey regarding participants' sexual life was conducted using the International Index of Erectile Function 5 (IIEF-5). The researchers administered the sexual function questionnaire (IIEF-5) in a separate and private space. The IIEF-5 is composed of 5 questions, each of which is scored from 0 to 5 points. A higher total score indicates better erectile function.

Sperm specimens were obtained by masturbation after 2-7 days of abstinence. After the specimens were acquired, the laboratory personnel immediately sent them to the temperature-controlled hospital laboratory to observe the appearance of semen, record the liquefaction time, measure the amount of semen, calculate the total number (modified Neubauer blood cell count plate) and concentration of sperm, and analyze the motility (wet sheet) and morphology (Diff-Quik staining) of sperm. The protocol and reference standards were derived from the standards in the fifth edition of the Laboratory Manual for the Examination and Processing of Human Semen.

Blood samples used for sex hormone testing were collected between 08:00 and 09:00. The levels of sex hormones were measured by chemiluminescence immunoassay analysis (Abbott Laboratories Ltd, Chicago, USA) and kits.

### 2.3. Statistical Analysis

All data were statistically analyzed using the SPSS 24.0 statistical software (IBM, Armonk, New York, USA). Data are presented as the mean ± standard deviation. Student's *t* test was used to analyze data with a normal distribution. Wilcoxon single-rank tests were conducted if the data did not conform to a normal distribution, and the chi-square test was used to assess rates and composition ratios. A value of *p* < 0.05 was considered statistically significant.

## 3. Results

### 3.1. Sexual Function, Sperm Quality, and Sex Hormones in Males with Epilepsy

A total of 44 young males with epilepsy (epilepsy group) and 30 healthy volunteers (control group) were recruited for this study. As shown in Table 1, there was no significant difference in age, body mass index, or sexual experience, but the marriage and procreation rates in patients with epilepsy were significantly lower than those in the control group (*p* < 0.01).

The sperm concentration, total sperm count, and IIEF-5 questionnaire score in the epilepsy group were lower than those in the control group (*p* < 0.05), while the sex hormone levels were not different (Table 2).

### 3.2. Effect of VPA and OXC on Sexual Function, Sperm Quality, and Sex Hormones in Males with Epilepsy

Twenty-one patients were treated with OXC (OXC group), and the average dosage was 706 ± 182 mg/day. Twenty-three patients received VPA (VPA group), and the mean dose was 1190 ± 295 mg/day. There was no significant difference between the two groups in terms of age, body mass index, duration of illness, marriage or procreation rate, sexual experience, number of epilepsy types, type of partial epilepsy, or seizure control (Table 3).

In patients treated with VPA for six months, the sperm concentration, total number of sperm, and percentage of sperm showing forward movement were significantly decreased (*p* < 0.05). The prolactin (PRL) level was significantly increased (*p* < 0.05), while no changes were observed in follicle stimulating hormone (FSH), luteinizing hormone (LH), testosterone (T), and estradiol (E2) levels and the IIEF-5 questionnaire score (Table 4).

After six months of OXC treatment, the FSH level was significantly increased compared with baseline, and the LH, T, PRL, and E2 levels were unchanged. The sperm quality and IIEF-5 questionnaire score also showed no significant changes (Table 5).

## 4. Discussion

Sexual and reproductive dysfunction is an important and common but often neglected aspect of epilepsy. At least 30% of men with epilepsy may suffer from sexual dysfunction [14]. Studies have shown that epilepsy itself as well as AEDs may affect reproductive endocrine function and sexual function, resulting in low fertility in men with epilepsy [15–21]. Our study results were in agreement with our previous research [6] showing that the marriage rate and fertility rate of young male patients with epilepsy were significantly lower than those of healthy young males.

Animal and clinical studies have suggested that epilepsy itself may affect sperm quality, sexual function, and sex hormones [22]. The decline in sperm quality and sexual function in men with epilepsy may be related to the disruption of hypothalamic pituitary axons by cerebral epileptiform discharges, resulting in changes in sex hormone levels [17]. For example, a decrease in the FSH level is not conducive to sperm maturation [23], and a chronic increase in the PRL level has a negative effect on male sexual function [24].

The negative impact of VPA on the sexual function of males with epilepsy has been recognized by some researchers [16]. Studies have shown that sexual dysfunction may be related to elevated PRL levels [8, 24]. The possible mechanism is as follows. First, high levels of PRL inhibit the secretion of hypothalamic gonadotropin-releasing hormone (GnRH), FSH, and LH, and the action of FSH is blocked. LH acts on the gonads, which leads to a decline in male sexual function. Second, high levels of PRL also inhibit the conversion of T into dihydrotestosterone (DHT), which has greater biological activity, and the reduction of active androgen levels in vivo has a negative effect on male sexual function [24].

In this study, we found that young male epilepsy patients taking VPA showed a marked decrease in sperm concentration, total number of sperm, and percentage of sperm with forward movement as well as a significant increase in PRL levels after 6 months of treatment. Previous studies have found that VPA may cause the testicular volume, sperm count, and sperm motility to decrease and cause the sperm deformity rate to increase [18, 25]. The mechanism by which VPA causes sperm damage in patients with epilepsy may involve the following three aspects. (1) VPA alters the level of sex hormones in patients. VPA has also been found to cause an increase in the level of PRL in mice and epilepsy patients [8, 18, 26]. High levels of PRL could inhibit the secretion of hypothalamic GnRH and pituitary gonadotropins (e.g., FSH and LH) as well as the conversion of T to DHT, which has greater biological activity. The decline in these hormones is not conducive to spermatogenesis and maturation [23, 27]. (2) VPA induces oxidative stress, which damages sperm cell DNA. Khan S et al. [28] found that VPA increases the expression of 8-oxo-deoxyguanosine (8-oxo-dG) in mouse testicular cells. 8-Oxo-dG is also a sensitive biomarker for DNA damage in human sperm cells [29], indicating that VPA can damage the sperm by damaging sperm cell DNA. (3) VPA reduces the carnitine level in humans. Among existing AEDs, VPA is considered the strongest carnitine-reducing agent, and long-term use will lead to lower levels of carnitine in the body [25, 30], while carnitine has a protective effect on sperm cells [31]. The reduced amount of carnitine causes a reduction in sperm quality. Although VPA can damage male sperm cells, studies have shown that this damage is reversible and can be reduced or eliminated after drug reduction or discontinuation [32].

Findings regarding the effects of OXC on sexual function in male patients with epilepsy have been inconsistent. Previous reports have shown that OXC may impair sexual function in male patients with epilepsy [16], but a study with a larger clinical sample by Luef et al. suggested that OXC can improve the sexual function of male epilepsy patients [12]. It is hypothesized that OXC may improve sexual function in male patients with epilepsy because it may increase potassium channel conductance and regulate high-potential activation of calcium channels, which may promote GnRH release from hypothalamic GnRH neurons [33, 34]. GnRH promotes pituitary secretion of LH, which is further stimulated by testicular synthesis of T to improve male sexual function [35]. In our study, erectile function scores showed a downward trend after VPA use and an upward trend after OXC use, but these trends were not statistically significant. This may be associated with the small sample size of our study. We also presume that VPA may reduce erectile function, while OXC may have improved erectile function.

To date, few studies have reported the effect of OXC on sperm quality and sex hormone levels. Isojarvi et al. [35] reported that the sperm concentration and sex hormone levels were normal in 18 OXC-treated men with epilepsy. Isojarvi et al. [36] also reported improvement of the serum sex hormone imbalance when carbamazepine (CBZ) was replaced by OXC. Cansu et al. [37] found no significant effect of OXC on testicular development in an animal study. Rattya et al. [9] indicated that the endocrine effects of OXC in men appeared to be dose-related because serum T, gonadotropin, and sex hormone-binding globulin (SHBG) levels were normal in patients treated with a daily dose of <900 mg OXC, but these serum hormone levels were elevated with a daily dose of ≥900 mg. In our study, it should be noted that a significantly increased FSH level was observed after OXC treatment. We also found that the sperm concentration and the total number and percentage of forward-moving sperm showed an increasing trend in the OXC group. Furthermore, the LH level was increased, whereas that of PRL was reduced, but the differences before and after treatment did not reach statistical significance. The above results indicated that OXC had no clear negative effect on male sperm cells and might be beneficial to the production of sperm cells. It is speculated that this may be because OXC can increase FSH and LH levels in humans, which is beneficial for spermatogenesis and maturation [23, 27].

The current study has several limitations. First, although sexual function, sperm quality, and sex hormones were evaluated, whether sexual function and sperm quality were associated with sex hormone disorders remains unknown. Second, due to the relatively small sample size, we failed to verify whether the above phenomenon was related to drug dosage. Thus, a well-designed study with a larger sample size is needed.

## 5. Conclusion

In conclusion, sexual function and sperm quality were reduced in young males with epilepsy. VPA may exert a negative effect on sexual function and sperm quality, whereas OXC has no harmful effect on sperm quality in young males with epilepsy.

## Figures and Tables

**Table 1:** Comparison of baseline data between the epilepsy group and the control group.

	Epilepsy group	Control group	*p*
Enrollment No. (patients)	44	30	—
Age (years old)^a^	26.8 ± 6.3	27.9 ± 4.6	NS
BMI (kg/m^2^)^a^	23.4 ± 2.0	24.0 ± 2.2	NS
Marriage rate (patients)^b^	19 (43%)	26 (87%)	<0.001
Fertility rate (patients)^b^	7 (16%)	21 (70%)	<0.001
Sexual life history (patients)^b^	38 (86%)	28 (93%)	NS

^a^Compared using the *t* test. ^b^Compared using the chi-square test.

**Table 2:** Comparison of semen quality, IIEF-5 questionnaire scores, and sex hormone levels between the epilepsy group and the control group.

	Epilepsy group	Control group	*p*
Sperm concentration (×10^6^/mL)	37.07 ± 6.54	41.77 ± 7.19	0.005
Total number of sperm (×10^6^/ejaculation)	128.93 ± 40.15	157.93 ± 41.23	0.004
Sperm percentage with forward movement	42.31 ± 3.93	43.97 ± 5.67	NS
Normal sperm percentage	2.56 ± 0.81	2.63 ± 0.92	NS
IIEF-5 questionnaire score (points)^a^	19.05 ± 2.65	21.70 ± 3.64	0.001
PRL level (mIU/L)	229.32 ± 61.76	213.55 ± 46.97	NS
FSH level (mIU/mL)	3.06 ± 0.93	3.21 ± 1.14	NS
LH level (mIU/mL)	2.69 ± 1.11	2.77 ± 1.27	NS
T level (nmol/L)	18.43 ± 5.58	18.26 ± 6.12	NS
E2 level (pmol/L)	111.16 ± 31.56	109.80 ± 33.10	NS

^a^The results were obtained from 38 sexually active epilepsy patients and 28 healthy volunteers who had sex.

**Table 3:** Comparison of baseline data between the VPA group and the OXC group.

	VPA group	OXC group	*p*
Enrollment No. (patients)	23	21	—
Age (years old)^a^	27.4 ± 6.5	26.2 ± 6.2	NS
BMI (kg/m^2^)^a^	23.4 ± 2.2	23.5 ± 1.9	NS
Course of disease (years)^a^	4.3 ± 2.7	4.7 ± 3.3	NS
Married (patients)^b^	10 (43%)	9 (43%)	NS
Married with children (patients)^b^	3 (13%)	4 (19%)	NS
Sexual life history (patients)^b^	20 (87%)	18 (86%)	NS
Simple partial seizure^b^	4 (17%)	3 (14%)	NS
Complex partial seizure^b^	14 (61%)	14 (67%)	NS
Partial to secondarily generalized seizure^b^	5 (22%)	4 (19%)	NS
Dosage (mg/day)	1190 ± 295	706 ± 182	—
No recurrence^b^	18 (78%)	16 (76%)	NS
Recurred 1-5 times^b^	4 (18%)	5 (24%)	NS
Recurred more than 5 times^b^	1 (4%)	0	NS

^a^Compared using the *t* test. ^b^Compared using the chi-square test.

**Table 4:** Comparison of semen quality, sexual function questionnaire scores, and sex hormone levels in the VPA group before and after treatment for 6 months.

	Before medication	After medication	*p*
Sperm concentration (×10^6^/mL)	37.70 ± 7.64	30.00 ± 9.17	0.003
Total number of sperm (×10^6^/ejaculation)	130.37 ± 43.81	97.29 ± 42.29	0.012
Sperm percentage with forward movement	41.95 ± 4.64	34.73 ± 8.15	0.001
Normal sperm percentage	2.58 ± 0.73	2.33 ± 0.79	NS
IIEF-5 questionnaire score (points)^a^	19.05 ± 2.82	18.05 ± 2.56	NS
PRL level (mIU/L)	226.33 ± 57.54	282.17 ± 103.67	0.029
FSH level (mIU/mL)	3.04 ± 0.86	2.79 ± 0.84	NS
LH level (mIU/mL)	2.78 ± 0.91	2.56 ± 0.92	NS
T level (nmol/L)	18.77 ± 6.22	18.74 ± 5.60	NS
E2 level (pmol/L)	109.61 ± 30.21	114.86 ± 26.57	NS

^a^The results were obtained from 20 epilepsy patients who had sex.

**Table 5:** Comparison of semen quality, sexual function questionnaire scores, and sex hormone levels in the OXC group before and after treatment for 6 months.

	Before medication	After medication	*p*
Sperm concentration (×10^6^/mL)	36.38 ± 5.19	37.57 ± .63	NS
Total number of sperm (×10^6^/ejaculation)	120.97 ± 39.51	125.62 ± 35.53	NS
Sperm percentage with forward movement	42.70 ± 3.03	44.66 ± 4.78	NS
Normal sperm percentage	2.53 ± 0.91	2.89 ± 0.56	NS
IIEF-5 questionnaire score (points)^a^	18.94 ± 2.56	20.00 ± 2.06	NS
PRL level (mIU/L)	232.60 ± 67.37	209.49 ± 73.10	NS
FSH level (mIU/mL)	3.08 ± 1.01	4.21 ± 1.74	0.014
LH level (mIU/mL)	2.60 ± 1.31	3.47 ± 1.65	NS
T level (nmol/L)	18.06 ± 4.89	19.85 ± 7.08	NS
E2 level (pmol/L)	112.86 ± 33.63	113.57 ± 21.52	NS

^a^The results were obtained from 18 epilepsy patients who had sex.

## Data Availability

All data needed to evaluate the conclusions in the paper are present in the paper.
